# Using oxygen dose histograms to quantify voxelised ultra-high dose rate (FLASH) effects in multiple radiation modalities

**DOI:** 10.1088/1361-6560/ac71ef

**Published:** 2022-06-08

**Authors:** Frank Van den Heuvel, Anna Vella, Francesca Fiorini, Mark Brooke, Mark Hill, Anderson Ryan, Tim Maughan, Amato Giaccia

**Affiliations:** 1 University of Oxford, Department of Oncology, Oxford, United Kingdom; 2 Radiation Oncology, Zuidwest Radiotherapeutic Institute, Vlissingen (Flushing), Zeeland, The Netherlands; 3 Oxford University Hospitals, Department of Hæmatology & Oncology, Oxford, United Kingdom; 4 Rutherford Cancer Centre Thames Valley, Reading, United Kingdom

**Keywords:** ultra high dose rate, oxygen, radiation biology

## Abstract

*Purpose.* To introduce a methodology to predict tissue sparing effects in pulsed ultra-high dose rate radiation exposures which could be included in a dose-effect prediction system or treatment planning system and to illustrate it by using three published experiments. *Methods and materials.* The proposed system formalises the variability of oxygen levels as an oxygen dose histogram (ODH), which provides an instantaneous oxygen level at a delivered dose. The histogram concept alleviates the need for a mechanistic approach. At each given oxygen level the oxygen fixation concept is used to calculate the change in DNA-damage induction compared to the fully hypoxic case. Using the ODH concept it is possible to estimate the effect even in the case of multiple pulses, partial oxygen depletion, and spatial oxygen depletion. The system is illustrated by applying it to the seminal results by Town (Nat. 1967) on cell cultures and the pre-clinical experiment on cognitive effects by Montay-Gruel *et al* (2017 *Radiother. Oncol.* **124** 365–9). *Results.* The proposed system predicts that a possible FLASH-effect depends on the initial oxygenation level in tissue, the total dose delivered, pulse length and pulse repetition rate. The magnitude of the FLASH-effect is the result of a redundant system, in that it will have the same specific value for a different combination of these dependencies. The cell culture data are well represented, while a correlation between the pre-clinical experiments and the calculated values is highly significant (*p* < 0.01). *Conclusions*. A system based only on oxygen related effects is able to quantify most of the effects currently observed in FLASH-radiation.

## Introduction

In ultra-high dose rate radiation, also known as *FLASH* radiation, the dose is delivered using pulsed radiation at rates which are of the order of 40 Gy s^−1^ or higher. The FLASH effect is generally associated with a reduction in the expected biological effectiveness compared to conventional dose rates and more specifically a reduction of normal tissue toxicities while maintaining local tumour control (Esplen *et al* 2020). There is ample evidence available that a FLASH effect exists and is measurable *in-vitro*. This comes from cell experiments mainly carried out in the late 1960s and early 1970s. In the latter experiment, ultra-high dose rate pulses (lasting a few nano-seconds) were used, delivered by either electrons or photons. Indeed, already in 1967 Town published results on high dose rate electron radiations (Town 1967), while Berry was able to use photons (Berry *et al* 1969) with very short pulses (of the order of nano-seconds), obtaining comparable results using the same cell types. More recent applications provide pre-clinical and even clinical applications (Bourhis *et al* 2019, Chabi *et al* 2020).

In addition, there are indications that tumour tissue is affected differently compared to normal tissue, showing a smaller or non-existent change in radiation effectiveness.

A possible mechanism for FLASH effects is the depletion of oxygen in the irradiated tissue, leading to a transient hypoxic environment, which provides a protective environment for part of the dose deposition, a possibility which was proposed by Ling (1975) as early as 1975 and again by Adrian *et al* (2019). More recently, Ling’s approach was taken up again by Petersson *et al* using more current knowledge on the impact of oxygen (Petersson *et al* 2020). However, this approach relies on oxygen depletion during the radiation pulse and does not take into account the protracted nature of oxygen depletion in combination with the dose deposition mechanism. Indeed the initial event takes place in a very short time frame. However, the time scale for DNA damage induction (and that of other macromolecules) via the indirect effect will be of the order of nanoseconds. IR damage to macromolecules, in general, is likely to play a much larger role in reactions with and subsequent depletion of oxygen than just DNA. It, therefore, relies on intricate knowledge of a very complex interacting system of events. In addition, the approach proposed by Petersson implies that the concept of oxygen enhancement (OER) is applicable in these very short time frames, which is a macroscopic concept which is being debated (see below). In addition, the extension to other modalities like protons and heavier charged particles is not straightforward from first principles.

The oxygen depletion mechanism has recently come under scrutiny. The criticism is based on a re-visitation of the possible rates with which oxygen can be depleted via the mechanism of radiolitic oxygen consumption. More specifically, Boscolo *et al* (2021) and Labarbe *et al* (2020) build models based on the radiolysis of water using different strategies. A common factor in these strategies is the attribution of oxygen depletion due to the irradiation events directly. Following these assumptions to their logical conclusion, they show that oxygen depletion is too slow and incomplete to account for the FLASH effect and the models worked out show that the experiments as generated by Adrian *et al* (2019) cannot be reproduced by a model based on radiolytic oxygen depletion model. Abolfath *et al* (2020), go a step further and uses a multi-stage simulation to model the DNA damage in FLASH conditions. Calculating the ionisation yields using track codes (Geant4-DNA) and subsequently a molecular dynamics simulation to identify the reactive oxygen species (ROS). Finally, the ROS can merge into non-reactive oxygen species (NROS). This simulation shows a more subtle dependency on oxygenation level by saturation of the NROS production in well-oxygenated environments.

Alternatively, biological processes are proposed, including changes in repair mechanisms, immune changes in the blood and others (Jin *et al* 2020). However, biological effects act on a time scale of the order of minutes to days and weeks, which would necessitate that differences in repair characteristics are already present in the various tissues. Or alternatively, the repair process is somehow impacted in a differential way in tumour cells compared to normal tissue by immune effects which also work on a different timescale. While these are interesting hypotheses, there are, to our knowledge, no quantifying models in the literature that could help predict the events. Finally, the data provided by Town in cells indicate that immune effects are likely not the primary mechanism, as cell cultures do not form part of an immune system and FLASH effects have been observed. These are therefore not addressed in this paper and using Occam’s razor we investigate the implications of an oxygen-based approach alone.

In this paper, we provide an ‘induced DNA-damage’ framework to estimate the impact of oxygen on FLASH-radiation, which incorporates the possibility of full modelling but does not rely on it. The proposed system is based on a previously developed algorithmically simple methodology (Van den Heuvel *et al* 2021) such that it can be readily incorporated into a planning system and/or inform experimenters to reduce confounding factors in experimental designs. It is constructed in such a way that it is readily expandable to other modalities like protons, alpha-particles and carbon ions.

The goal of this paper is therefore not to provide an explanation of the mechanism responsible for the FLASH-effect, but rather to provide a way to incorporate the effect into treatment planning based on our current experimental knowledge. For this, we use observations stemming from the *in-vitro* experiments. Specifically, in Town’s paper:

(i) A clear dependency on oxygenation is shown by comparing the survival curves in oxygenated and hypoxic cell cultures. Showing a dose threshold behaviour at about 10 Gy of delivered dose.
(ii) A difference in dose-dependency between doses delivered in a single pulse and those using two pulses.

It should be noted that Town’s paper is not without its problems. There seems to be a discrepancy in the results of the two experiments. Where one shows a very strong FLASH effect such that flat response is exhibited. The other experiment still shows a FLASH effect but the data seems to be more realistic, it is issues like these that provide a rationale for a model-based approach.

## Methods and materials

### Damage model

**Figure 1.**
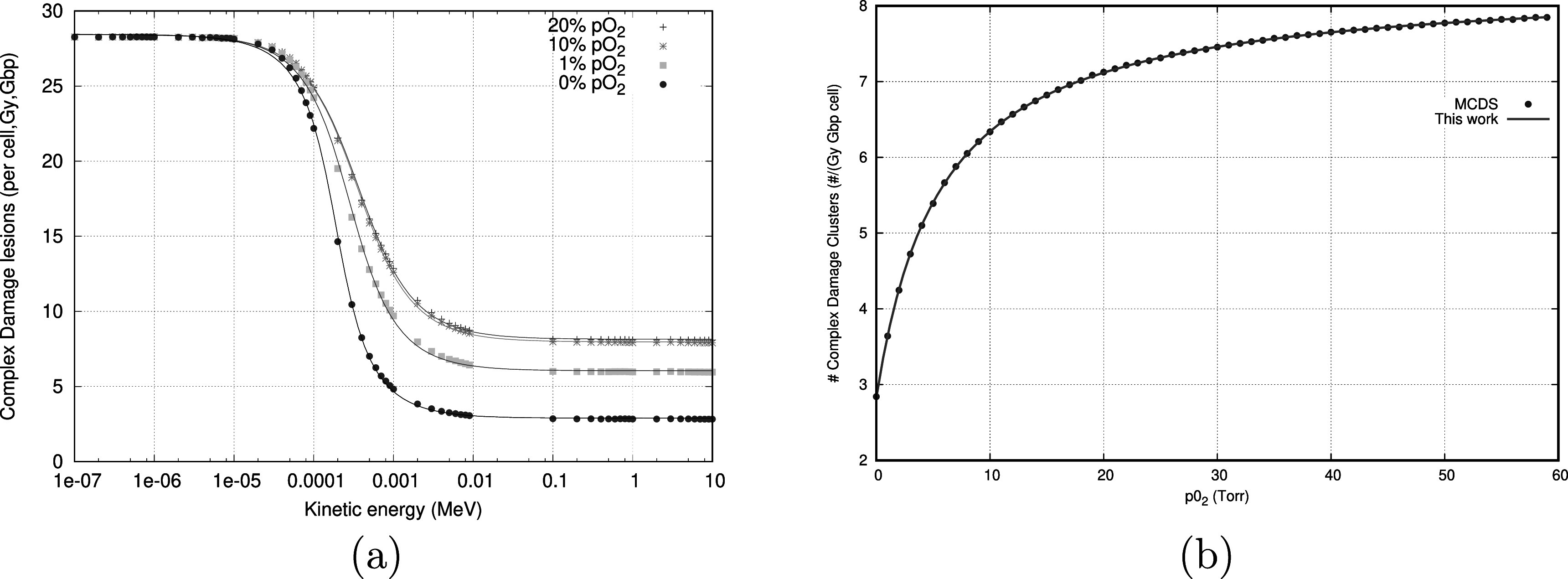
Figures illustrating the energy dependence of DNA-damage induction (left), and the oxygenation level dependence (right).

In this model, we quantify the impact of radiation on living cells in terms of DNA-damage induced. More specifically, complex DNA-damage clusters are defined as at least as complex as double-strand breaks. This process, which has been supported extensively by cell data, predicts the differences in treatment modalities well and has been used to incorporate some biological properties into treatment planning (Sato *et al* 2009, Stewart *et al* 2011, Van den Heuvel 2014). This methodology has been shown to be linear with respect to dose to at least a few 100 Gy (Stewart *et al* 2015). We define a damage map as the quantification of the number of damage clusters generated per cell, per Giga base-pair (Gbp) and per Gy in a given dose voxel ${M}_{d}.$ Indications exist that cell survival is related to the yield of clustered lesions, these lesions include DSB with additional strand breaks and/or base damage within 10 base pairs (Hall and Giaccia 2019). Figure 1(a) illustrates the dependency of ${M}_{d}$ as a function of particle energy for electrons.

### Impact of oxygen on DNA-damage

The impact of oxygen in radiation biology has been studied extensively in the past and the mechanism is well established. More specifically the mechanism of oxygen fixation of DNA-damage is the current canonical mechanism. In this model, the presence of highly reactive oxygen fixates damage introduced by ionising radiation. This process of fixation competes with chemical repair processes affected by, among others, thiols. Empirically, the amount of oxygen available governs the efficacy of the fixation process as shown overwhelmingly in many publications (Gray *et al* 1953, Brown 2007, Wardman 2015). It is therefore reasonable to model oxygen as a limited resource, which allows quantifying the effect (Kepner 2010).

In a separate paper, we showed that oxygenation effects, during irradiation by charged particles, can be described in terms of a linear transformation of the microscopic saturation equation (Van den Heuvel *et al* 2021), which we present here in a shortened version.

An oxygen fixation mechanism is modelled as a Ligand-Receptor system where the dependence of the concentration of available Ligands (i.e. oxygen concentration) follows an expression of the form:

\begin{eqnarray*}\displaystyle \frac{[{M}_{T}][{{\mathrm{O}}}_{2}]}{{K}_{D}+[{{\mathrm{O}}}_{2}]},\end{eqnarray*}

where ${K}_{D}$ is the dissociation constant and ${M}_{T}$ the maximal amount of oxygen fixation sites. The dissociation constant is effective due to the competitive nature of oxygen fixation with thiol repair. This can be re-written as a function of oxygen concentration, quantifying the impact of oxygen on the number of repaired damage sites:

\begin{eqnarray*}F\left(\left[{{\mathrm{O}}}_{2}\right]\right)=\displaystyle \frac{[{{\mathrm{O}}}_{2}]}{{q}_{1}+{q}_{2}[{{\mathrm{O}}}_{2}]},\end{eqnarray*}

where the parameters ${q}_{1}={K}_{D}/{M}_{T}$ and ${q}_{2}=1/{M}_{T}$ are respectively unitless and an inverse concentration of fixation sites.

In an environment where there is a variety of complex damage, the expression in equation (2) only describes a relative change. If, for example, a large amount of irreparable damage is present then the impact of oxygen fixation will be small as oxygen is used to fixate damage that is already irreparable. Van den Heuvel *et al* (2021) argued that this can be described as a linear transformation of Formula 2.

With $a(y)$ a damage factor and $b(y)$ a minimal level of damage (i.e. damage present without the availability of oxygen) whose units are a number of double-strand breaks per Gy, per Giga base pair (Gbp), per cell and which depend on the kinetic energy ($y$) of the interacting particle. The linear transform determines the changed damage impact (${M}_{d}(y,[{{\mathrm{O}}}_{2}])$) which has the form of:

\begin{eqnarray*}{M}_{d}\left(y,\left[{{\mathrm{O}}}_{2}\right]\right)=a\left(y\right)F\left(\left[{{\mathrm{O}}}_{2}\right]\right)+b\left(y\right),\end{eqnarray*}

where *b*(*y*) can be determined by a model, micro dosimetric Monte Carlo simulations, or measured data providing damage levels (${M}_{d}\left(y,0\right)$) in fully hypoxic conditions (Van den Heuvel 2014). In a mono-energetic environment this can be reduced to the following expression:

\begin{eqnarray*}\displaystyle \frac{{C}_{1}[{{\mathrm{O}}}_{2}]+{C}_{2}}{[{{\mathrm{O}}}_{2}]+{C}_{3}},\end{eqnarray*}

where the fitting parameters ${C}_{1},$ ${C}_{2},$ and ${C}_{3}$ are now defined for convenient curve fitting rather than physical meaning. Equation (4)’s simplicity makes it very suitable to incorporate in a standard Monte Carlo engine to include oxygen effect information in a dose deposition run.

In the special case of a standard photon (100 kVp—20 MV) and (100 keV—20 MeV) electron based irradiations, the oxygen effect is very weakly dependent on the energy (see figure 1(a)) and single energy can be chosen to calculate the oxygen dependence in a poly-energetic electron or photon beam^5^ 
^5^
In this paper we consider electron and photon-based irradiation to be equivalent, as photon therapy can be viewed as dose deposition by secondary electrons. This is because ionisations due to electrons (in Megavoltage photon beams) outperform photon ionisations by a factor of 10^5^..

Below, parameters for oxygenation (in Torr) dependence of 2 MeV electrons are provided and are the fit parameters used in figure 1(b). These will be used in the example section applying equation (5)

\begin{eqnarray*}F([{{\mathrm{O}}}_{2}])=\displaystyle \frac{{C}_{1}[{{\mathrm{O}}}_{2}]+{C}_{2}}{[{{\mathrm{O}}}_{2}]+{C}_{3}}.\end{eqnarray*}

\begin{eqnarray*}\begin{array}{l}{C}_{1}=8.334\pm 0.002\\ {C}_{2}=15.99\pm 0.07\\ {C}_{3}=5.67\pm 0.02.\end{array}\end{eqnarray*}

(a) Energy dependence of induction of double-strand breaks (or more complex) as a function of electron energies at different oxygen levels (0, 7.6, 76, and 152 Torr pO2) as calculated by MCDS, the full lines are model fits as proposed by Van den Heuvel (2014) and Stewart (Stewart *et al* 2015).
(b) Applying equation (5) for electrons with a kinetic energy of 2 MeV. Lines are this work. Points are from MCDS simulations. Note that the largest variation of damage induction occurs in the clinically relevant region of 0–20 Torr pO_2_.

In radiobiology, the quantification of partial oxygen pressure is frequently reported as a percentage. In clinical practice tissue oxygenation levels are reported in millimetres of mercury pressure (mmHg). Which in this case is a better measure as levels in human tissue are of the order of 20mmHg (pO_2_) in well-oxygenated tissue and 100 mmHg (pO_2_) in arterial blood. In the remainder of the article, we will be using the more precise unit of Torr (1 Torr $\simeq $ 1 mmHg).

### Oxygen depletion

Whillans and Rauth have measured oxygen depletion as a function of delivered dose and proposed a linear model for depletion (Whillans and Rauth 1980):

\begin{eqnarray*}[{{\mathrm{O}}}_{2}]={[{{\mathrm{O}}}_{2}]}_{0}-{d}R[{{\mathrm{O}}}_{2}].\end{eqnarray*}

The initial partial pressure ${[{{\mathrm{O}}}_{2}]}_{0}$ is expressed in terms of [pO_2_ Torr], dose $d$ in [Gy], which makes the depletion rate $R$ in terms of [pO_2_ Torr/Gy]. Rates between 0.21 and 0.42 Torr Gy^−1^ have been observed, it should also be noted that this result stems from standard ^60^Co radiation at about 1 Gy min^−1^. It is difficult to transpose the latter results to an ultra-high dose rate regimen where we would apply this depletion within the fine pico-second structure of a pulse. This is because oxygen depletion is not instantaneous, but rather the result of a cascading process initiated by the generation of radicals in a complex process taking several nanoseconds (ns).

Indeed, Clifton Ching Ling (1975) proposed a theoretical analysis of oxygen depletion in an ultra-high dose rate regimen by positing a depletion by the interaction of the available oxygen with any radiation-induced lesion (including DNA-lesions). Interestingly, he decoupled the direct ionisation process from the oxygen depletion process, considering the first as a trigger of a cascade process (which includes the generation of indirect damage). The interaction of the available oxygen, with any induced species, is modelled as a second order reaction. The time dependent concentration, starting from an initial concentration ${[{{\mathrm{O}}}_{2}]}_{0}$ then becomes:

\begin{eqnarray*}\left[{{\mathrm{O}}}_{2}\left(t\right)\right]=\displaystyle \frac{\left\{GD{\left[{{\mathrm{O}}}_{2}\right]}_{0}-{\left[{{\mathrm{O}}}_{2}\right]}_{0}^{2}\right\}{e}^{\lambda \left({\left[{{\mathrm{O}}}_{2}\right]}_{0}-GD\right)t}}{GD-{\left[{{\mathrm{O}}}_{2}\right]}_{0}{e}^{\lambda \left({\left[{{\mathrm{O}}}_{2}\right]}_{0}-GD\right)t}}.\end{eqnarray*}

The quantity denoted by $\lambda $ is the binding rate of the oxygen to any lesion. The delivered dose $D$ impacts the medium by generating a concentration of radiation induced species with a rate of $G$ per dose unit.

### Pulsed dose delivery

In standard medical linear accelerators, pulse lengths are typically of the order of a few s and consist of closely spaced pico-second pulses. Due to these orders of magnitude it is convenient to define a new quantity, the instantaneous dose rate $\dot{D},$ expressed in cGy/ns. The area under a pulse represented in a dose rate versus time graph is the total dose delivered in a single pulse. In short total dose $D=L\times \dot{D},$ with *L* being the pulse length. In the case where the dose rate is not constant (i.e. the pulse shape is not rectangular):

\begin{eqnarray*}D=\displaystyle {\int }_{0}^{L}\dot{D}\left(t\right)dt.\end{eqnarray*}

This approach works well when the pulse lengths are of the order of the oxygen depletion times, but seems to be flawed when very short pulses are used (as in the case of the experiments by Berry (Berry *et al* 1969) and Ling (1975)). But also when other pulse sequences are used for instance in quasi-continuous exposure where nano-second pulses a few nano-seconds apart are delivered (Darafsheh *et al* 2020). It seems therefore that the notion of oxygen depletion during a pulse is not a useful model in all cases.

### Oxygenation dose histogram

Rather than trying to model the complete process in a time-dependent manner, an abstraction is made. We observe that there is a range of oxygenation levels present during the dose deposition process, which is a protracted process in itself. Both oxygen depletion and the dose deposition process take place in a time resolution in the range of a few nanoseconds.

It stands to reason that we can subdivide the process into delivered dose quanta, each at a given oxygenation level. It follows that we can abstract this by creating a histogram, where we set out the delivered dose as a function of the oxygenation levels present throughout the dose delivery. As it is a histogram we also lose positional information as well as synthesising the effect of the pulsed delivery. The only information is the instantaneous dose deposition under given oxygenation conditions wherever and whenever (within the pulse) this occurs. This subtle difference resolves the problem that the oxygenation fixation deals with oxygen removal in the immediate vicinity of the DNA-strand (i.e. a few nanometers) while oxygen depletion takes place over the whole of the irradiated volume. Not only that but both geometric heterogeneous oxygen depletion and incomplete depletion can be modelled. The surface under this *Oxygenation Dose Histogram* (ODH) is the total delivered dose.

**Figure 2.**
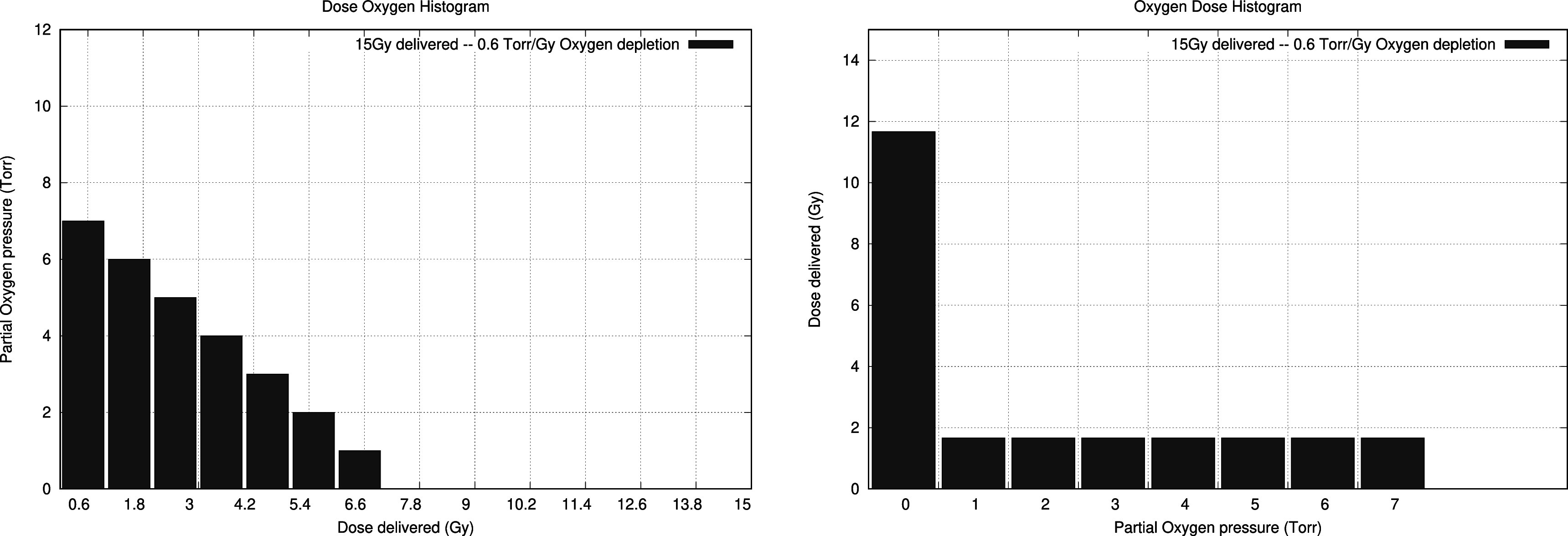
Left: An example of a DOH where we assume the oxygen depletion occurs in a linear fashion within a single pulse with an initial oxygenation level of 7 Torr (pO_2_). Right, the ODH representation, where the axes are switched. In this particular example of a linear depletion (i.e. a constant depletion rate) all bins have the same height (the depletion rate/bin width) except the final one.

The generation of these histograms can be informed either by Monte Carlo simulations of the oxygen depletion model, an analytical expression, or by experimental results. All of which can generate the *inverse* ODH which we coin, in Homerian fashion, DOH (dose oxygen histogram). An example where we propose a linear dose depletion of 0.6 Torr Gy^−1^ while delivering 15 Gy is shown in figure 2. It is important to note that the latter is a specific way of generating the ODH, but not the only one, nor the correct one. In this ODH a substantial part of the dose is delivered in complete hypoxia.

The model presented in equations (3) and (5) can be used to quantify the impact of the oxygen environment in conditions which are related to the FLASH-effect. The ODH is converted to total complex damage count ${M}_{D}$ which can readily be compared to a damage count in a case where no change in oxygenation takes place (${M}_{{D}_{0}}$). The ratio ${M}_{D}/{M}_{{D}_{0}}$ then provides a quantification of the FLASH effect which is always lower than 1, and therefore is a sparing effect.

An interesting property of ODHs, in contrast with the more intuitive DOH, is that they are additive, facilitating the implementation of pulsed treatments with partial depletion and/or incomplete re-oxygenation. Indeed, if a subsequent pulse with a different oxygenation signature is delivered we can simply add both ODH together and perform the calculation. A special case is when complete (local) re-oxygenation occurs between pulses: the histogram of a single pulse can be added to itself and therefore is multiplied by the number of pulses.

### Damage implications of FLASH experiments

Next, we illustrate the effect of our model on the generation of DNA-damage in the presence of different initial levels of oxygen and with variable pulse lengths (i.e. different total doses). The modality chosen here is an electron beam with a median depositing energy of 2 MeV. This is commensurate with most experiments found in the literature. Due to the flat response of DNA-damage with respect to energy, the use of a mono-energetic approximation is reasonable as can be observed from figure 1(b). Indeed, we repeated our calculations at different energy levels varying from 100 keV to 10 MeV, which yielded identical results (data not shown).

As an example we deliver ultra-high dose rate irradiation of 15 Gy using a single pulse with a width of 3.4 s. In a first approximation, we use a linear inverse ODH. Starting at the initial oxygenation level and descending linearly to full hypoxia. We elect to use the steepest depletion rate reported by Whillams (e.g. 0.42 Torr Gy^−1^). Using equation (5) we calculate the expected number of complex damage clusters per cell and per giga-base pair (Gbp).

## Results

### General observations

#### A major mechanism for the FLASH effect

**Figure 3.**
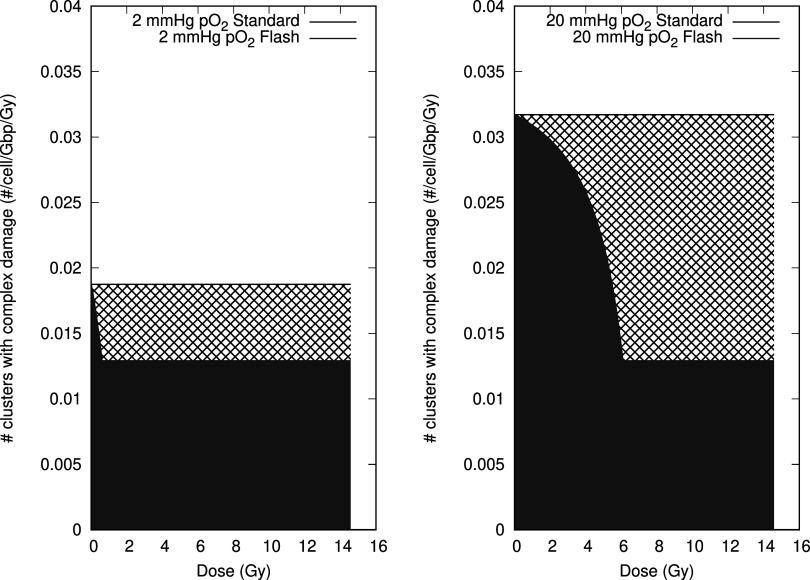
The difference in expected effects depends on the amount of initial oxygenation. Both 2 Torr pO_2_ (left) and 20 Torr pO_2_ (right) show a reduction in effectiveness. But the first less so than the latter. Also note that if one decreases the dose (i.e. the pulse length), the difference becomes smaller and can even reverse (see figure 4). This shows a clear non-linear effect due to the co-variance of dose and initial pressure variables. The generated lesions are per cell, Gbp, and Gy.

Figure 3 illustrates the major effect of flash therapy. Due to differences in initial oxygenation in cells, the induced damage differs depending on how much dose is delivered. The grey surface shows the amount of damage generated if the oxygenation is constant throughout the irradiation. The magenta surface area is always smaller than the grey one. The ratio of magenta versus grey is defined as the sparing effect.

#### Initial oxygenation

For simplicity’s sake, we keep to a single-pulse application using a pulse length of 3.4 s, delivering 15 Gy an application which is known to exhibit the FLASH-effect and is illustrated in figure 3. It calculates the DNA-damage induced before biological repair takes place. We allow the initial oxygenation to vary between 0 Torr pO_2_ and 20 Torr pO_2_. In figure 5 we show the sparing effect by comparing the damage inflicted in a flash regimen to that generated in normal conditions.

**Figure 4.**
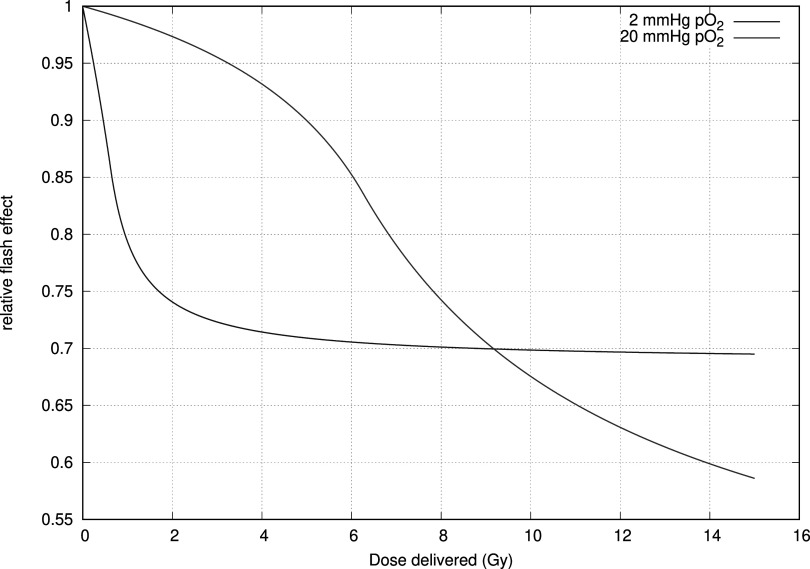
Estimating the flash effect at 2 different initial oxygenation levels. To illustrate the difference between fully oxygenated tissue and moderately hypoxic values, which could be present in tumour tissue. Depending on the dose delivered the sparing occurs to a larger extent in one tissue over the other.

Figure 4 shows how the FLASH effect depends on the dose delivered in flash mode for two different initial oxygenation levels. We vary the delivered dose by varying the beam on time within the pulse. The figure shows that there is a dose level where the FLASH effect is larger for the lower oxygenation level. At about 10 Gy there is a crossover and the well-oxygenated tissue exhibits a larger amount of sparing. In addition, the limit in maximal sparing is different for both cases.

#### Experiments in well-oxygenated environments

In the case of cell experiments, the initial oxygenation level is not always reported accurately. In a majority of the experiments, it is reasonable to assume an atmospheric oxygenation level which, in normal circumstances, is about 20% availability of oxygen in the air.

**Figure 5.**
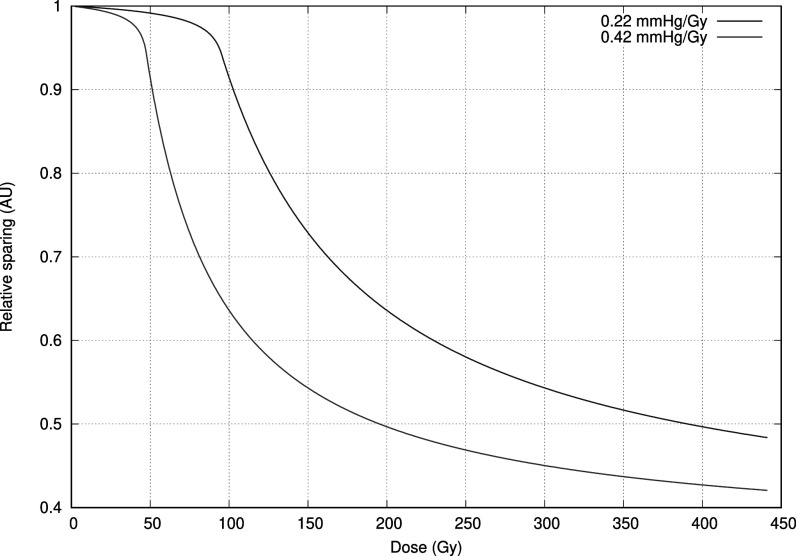
At oxygenation levels comparable to atmospheric conditions, the doses needed for cells to exhibit the flash effect are exceedingly high. We present the extremes in oxygen depletion rates.

In figure 5 we show the relative maximal sparing effect for different doses delivered in 3.4 s, normalised to the condition where no oxygen depletion takes place. We show the effect of the extremes in the reported oxygen depletion rates (i.e. 0.22 Torr Gy^−1^ and 0.42 Torr Gy^−1^).

**Figure 6.**
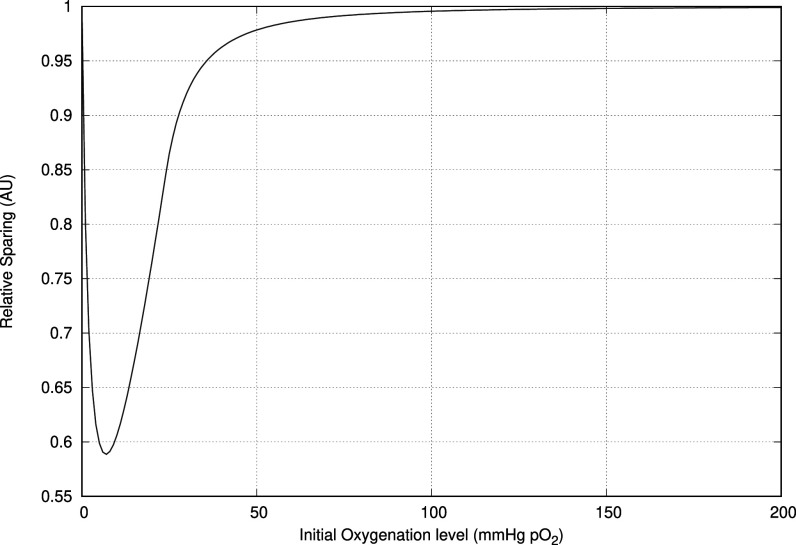
Graph showing the dependency of initial oxygenation and level of sparing effect. This is for a 15 Gy dose delivery in a pulse of 3.4 *μ*s. It is clear that in some cases small variations in oxygenation can have a major impact on the sparing effect.

From the results of the cell culture, we find that the initial oxygenation level (i.e. the oxygenation level at the beginning of the pulse), plays an important role in determining whether a FLASH protective effect takes place and also how large this effect is. Using our standard 3.4 s pulse delivering 15 Gy, we vary the initial oxygenation level. Figure 6 shows that there is a relatively narrow window in which the FLASH effect can take place. In addition, the response as a function of partial pressure can be quite steep leading to large uncertainties.

### Flash applications

In this section, we describe the application of this model to experiments in an Ultra High Dose Rated (UHDR) environment. Most published data use electrons as a modality. The experiments were chosen because they had an adequate description of the environmental conditions and consisted of data points exhibiting both FLASH and non-FLASH outcomes. These were the original experiment by (Town 1967), and the animal experiments by Montay-Gruel (Montay-Gruel *et al* 2017).

#### Re-creation of a cell based experiment

In 1967 a seminal paper on high dose electron radiation described a protective effect in cells by (Town 1967). In this paper, an effect was shown when using pulses of 1.2 *μ*s. The dose was varied from 0.5 to 45 Gy. In the experiment, the dose was delivered to HeLa cell cultures in single- and double-pulses. The single-pulse experiments exhibited a FLASH effect, showing a hockey stick response as a function of the dose delivered. In this paper, the data from the experiment was digitised. An ODH was calculated starting from a linear depletion model (i.o.w a linear DOH). The methodology outlined above yielded FLASH sparing values at each dose. Using the additivity of the ODHs the same was done for the double-pulsed deliveries. The survival fraction (SF) for the double pulsed data was corrected with the FLASH sparing value (*F*) by decreasing the number of cells killed.

\begin{eqnarray*}S{F}_{corr}=1-(1-S{F}_{meas})\times F.\end{eqnarray*}

**Figure 7.**
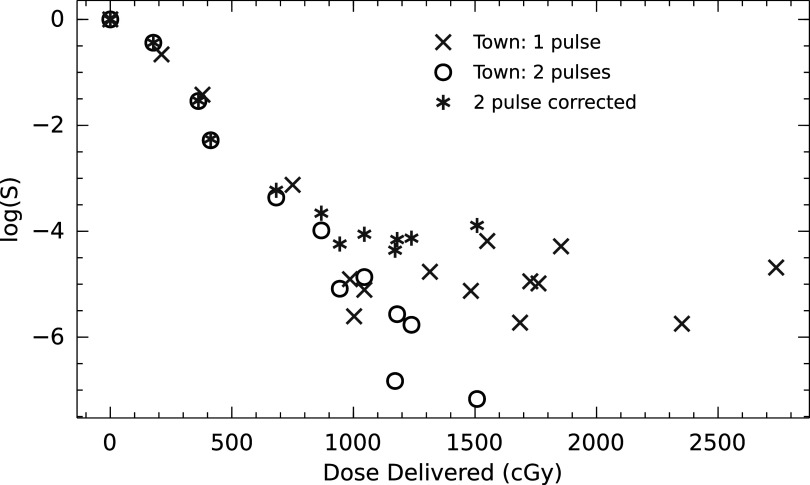
(a) Digitised data from Town (Town 1967), Sparing ratio, comparing single-pulse (*x*) to double-pulse (o), Double-pulsed corrected points (*) (i.e. surviving fractions are corrected with a FLASH Sparing Ratio) show the same behaviour as the single pulsed points.

The figure 7 shows the best agreement when choosing 15.0 Torr pO_2_/Gy as the depletion rate starting at 152 Torr (20% pO_2_) oxygenation, s depletion rate much steeper compared to the 0.42 Torr Gy^−1^ as measured by Whillans (Whillans and Rauth 1980). Something which was also noted by Town.

#### Re-creation of a pre-clinical experiment

In this section we apply our model to the results from the paper: ‘Irradiation in a FLASH: unique sparing of memory in mice after whole brain irradiation with dose rates above 100 Gy s^−1^’ by Montay-Gruel *et al* (2017).

In this paper, the authors subjected a cohort of mice to brain irradiations with different dose rates and tested the mental capacities by estimating the Recognition Ratio in an object recognition test. We quote:

Evaluation of the Recognition Ratio (RR) two months post-irradiation [was performed] for groups of mice that received sham irradiation (control) and 10 Gy (Whole Beam Irradiation) with a dose rate of 0.1, 1.0, 3, 10, 20, 30, 60, 100, or 500 Gy s^−1^, or with a single 1.8 s electron pulse (1 Pulse).

We make the assumption that full re-oxygenation between pulses occurs which, in this case, are 10 ms apart. So only the dose within a single pulse is of interest. This is a reasonable assumption as the original cell-based experiments showed that the effect was minimal when delivering the treatment in 2 pulses 2.5 ms apart. In the ODH formalism, this is the special case where the ODHs of all pulses are identical. In order to apply our model, we need to convert from the Gy/s notation to a more refined cGy/ns within a single pulse. This is done as follows:

(i) Divide the Gy/s expression by the pulse repetition frequency to get the dose per pulse (DPP): so here $\tfrac{x{\mathrm{Gy}}\,{{\mathrm{s}}}^{-{\mathrm{1}}}}{100}$
(ii) Calculate the relative sparing effect using effective depletion rates as gleaned from the *in-vitro* experiments (Town 1967) and initial oxygenation rates which should be of the order of 20 Torr pO2 for healthy tissue or lower.

**Figure 8.**
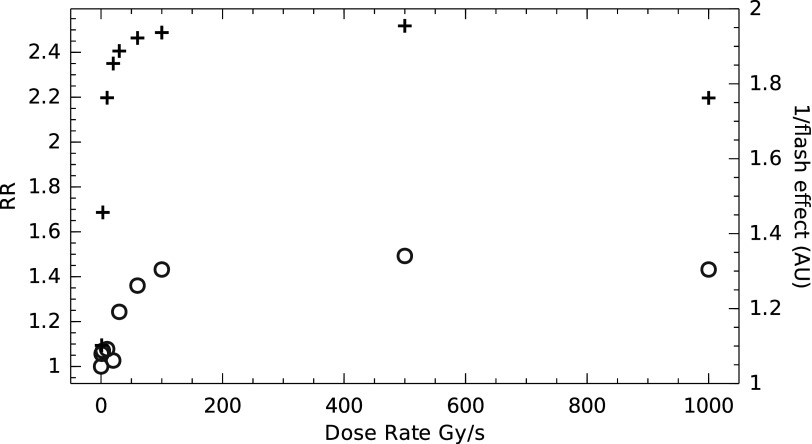
The open circles (o) are recognition rates (left *Y*-axis) as a function of dose rate. The plus signs (+) are the inverse of the FLASH effect ratio (right *Y*-axis). Assumptions are that the ODHs for cell cultures are validly generated with 15.5 Torr Gy^−1^ and 20 Torr pO2 oxygenation of the brain. The single-pulse data using a 1.8 m pulse is indicated as 1000 Gy s^−1^.

We then calculate the sparing factor in the same manner as before and invert it, yielding figure 8, showing a correlation when choosing an initial oxygenation level of 20 Torr pO_2_. The inversion is needed as we expect the Recognition Rate to increase with increased sparing.

The correlation, quantified using Spearman ranking, provides a $\rho $ = 0.881 which even with the limited number of points (10) is significant at *p* < 0.01, the latter correlates with a critical Spearman Ranking correlation coefficient ${\rho }_{c}$ = 0.794 as provided by (Zar 1972). The correlation is robust for a range of combinations of oxygen depletion rates and initial oxygenation levels.

### Recreation of other cell experiments

Recently, other cell experiments with modern accelerators and proton machines and new cell lines were published. The analysis of which would make this paper too large. In addition, many of the experiments are aimed at showing that a FLASH effect either exists or does not but do not investigate the parameters that show the transition region or which have an ill-defined approach to dose rates delivered (mix of single and multiple pulses). For illustration purposes, we have reviewed such an experiment and compared it to our methodology. The paper reviewed is a proton experiment using high-intensity Laser generated proton beams by Doria *et al* (2012) which in turn is a result of the PhD thesis by (Fiorini 2012), this can be found in an addendum. In addition, we reviewed an investigation on the impact of oxygen by Adrian *et al* (). Unfortunately, the data provided in the paper alone did not allow us to perform an in-depth analysis, specifically the fact that every dose point consisted of irradiation using a different number of pulses.

## Discussion

Our work provides some interesting corollaries, which impact the applicability of FLASH therapy in clinical practice.

(i) The level of initial oxygenation in a given tissue is critical to the magnitude of the FLASH effect.
(ii) There is a dose-dependency of the FLASH-effect. If the dose is too low, no FLASH effect exists.
(iii) Mathematically, the FLASH-effect is considered redundant in its modelling parameters. This means that a single result can be obtained by different combinations of specific parameters (oxygenation level, Dose rate, total dose delivered).

Indeed the existence of a threshold dose for the FLASH-effect restricts the applicability and also makes the physical implementation difficult, for instance when using scanned proton beams or when combining beams from different angles. In addition, the lower limit to the FLASH effect still exhibits a biological effect commensurate with about 50% of the original dose. This combined with the threshold dose means that the tissue we want to spare can still receive a significant effective dose. The dependency on the initial oxygenation is another major issue as it is difficult to measure in a clinical situation. Moreover, the dependency can be quite steep at some levels, giving rise to major uncertainties. This will therefore necessitate a methodology that is able to more accurately measure the oxygenation levels *in-vivo*. At the current level of clinical technology, this could be a major hurdle. A first step would be to apply a FLASH technique using standard dose prescriptions and take any reduction in normal tissue complications as a boon. In a further step, oxygenation would need to be measured and adjustments to treatment parameters would take into account the accuracy with which we can determine oxygen levels. Again, a framework as proposed here is helpful to determine the accuracy needed. A measurement option to determine *in-vivo* oxygen levels could be the use of MR-imaging, which is now being rolled out using MR-linacs (Raaijmakers *et al* 2005, Mutic and Dempsey 2014), using diffusion weighting as a marker or somewhat more direct blood oxygenation level-dependent (BOLD)-MRI, or, alternatively Oxygen enhanced (OE)-MRI. The former would only serve as an indicator as it is assumed that lower diffusion would be associated with a decrease in oxygenation and could indicate where to expect a FLASH-effect to be present and where not. Quantification of the effect would be more difficult. In the case of BOLD-MRI and OE-MRI quantification, both rely on the use of pure oxygen or hyperoxic carbogen, complicating issues in a radiation therapy environment due to logistics and the fact that it is a radiation sensitizer. Many questions remain as the difference in relaxation rates are not quite linear with the oxygenation in Torr pO_2_ (O’Connor *et al* 2019). Alternatively, positron emission tomography (PET) also claims to be able to determine oxygenation levels and has, at least theoretically, been proposed for use in radiation therapy (Van den Heuvel *et al* 2013). Here also most methods of determining the oxygenation levels are indirect and necessitate at least the knowledge of a base line oxygenation level. Only when using 15O isotopes as an agent is it possible to provide direct quantification. Finally, the use of electron spin resonance (ESR) seems to have an accuracy comparable to what we might need and can provide direct quantification in terms of Torr pO2. Unfortunately, to our knowledge, this has not been translated into clinical use in humans but has been investigated in a pre-clinical environment (Hashem *et al* 2015).

An overview of these techniques can be found in a review by Tretter *et al* (2020).

An approach to include this framework in a planning system would then be as follows:

(i) For every voxel, in a 3D dose deposition matrix, the dose and the energy spectrum are calculated.
(ii) For each voxel, the dose rate characteristics are calculated, depending on beam characteristics (pulse architecture, and beam arrangement).
(iii) For each voxel, an estimate of the initial oxygenation conditions needs to be quantified.
(iv) Generate the ODH for each voxel.
(v) Calculate Damage levels using the above-developed framework.

This workflow has been implemented using the free software treatment planning system: matRAD developed at Heidelberg (Germany) (Wieser *et al* 2018) and was presented at the ESTRO conference in 2020 (Van den Heuvel *et al* 2020a)

The approach outlined in this paper is based on a number of assumptions with respect to the oxygen depletion and fixation processes.

(i) Oxygen depletion is a statistical process and is not modelled as a localised phenomenon related to damage induction. In other words, the induction of DNA damage is not coupled with oxygen depletion. However, given the discrepancy in oxygen depletion rates between experimental data from FLASH-radiation (Town) and conventional (Whillams) it is still necessary to consider FLASH effects on oxygenation as a localised phenomenon giving rise to pockets of relative hypoxia.
(ii) The impact of oxygen on DNA-damage induction is a microscopic process, whereby oxygen is used up in a very small volume with a diameter of a few nanometers.

When estimating the ODHs in this paper we assumed an effective linear depletion rate. This is likely not correct but does give adequate results. In future implementations, we expect that ODHs will be determined using more sophisticated means where the various processes, like damage induction and diffusion of radicals in cells, are simulated. These will result in very heterogeneous oxygen distributions in time and space, which can be captured using this formalism. It might even well be that results from water radiolysis are not applicable to cells as the diffusion coefficients can be quite different.

In more recent experiments the depletion of oxygen by radiation was found to be incomplete and a residual amount of oxygenation remained as the oxygen depletion at very high dose rates showed a limited efficiency. Jansen *et al*, have looked at the radiochemistry, noting that at higher dose rates the oxygen consumption seems to decrease, which they attribute to a large number of radicals reacting with/among themselves rather than with oxygen (Jansen *et al* 2021). This observation is not irreconcilable with our methodology as can be seen in figure 1(b), where a substantial response difference still exists between 20 Torr pO_2_ and some of the lower values.

The results obtained in this paper only consider electrons, photons, and protons. These can be expanded readily to other modalities like protons, alpha-particles and Carbon ions. Indeed the mathematical damage model shows that the same approach for different particles is valid and mathematically similar, but at different energy levels, and the oxygenation model is identical. However, the simplification that we can use the oxygenation characteristics from a single energy is no longer valid. In those cases, part of the energy deposition now occurs in the steep region of the curves shown in figure 5. This can be resolved using a weighted sum of the spectral contributions to the damage. Together with the oxygen impact model (i.e. as in equation (5)), one can calculate the impact of spectral composition for each type of particle. A short calculation shows that for protons the single energy approximation is still reasonable, which is illustrated in the addendum. In the formalism introduced here, we imply a strong connection between the FLASH sparing effect and the Oxygen Enhancement Ratio. A direct corollary is that for particles with a small OER the FLASH effect is diminished. This is because in this model the FLASH effect is generated by a difference in damage induction at different oxygen levels. If the OER is small then the difference in damage induction is diminished. In a previous paper, we show that there is almost no difference in OER between the spread-out Bragg peak (SOBP) region and the upstream region in a clinical proton beam (Van den Heuvel *et al* 2021). We, therefore, predict that there will be no measurable difference with regard to the FLASH effect.

For $\alpha $-particles and carbon particles this is likely not the case. There the oxygen effect is lower in the SOBP and this model predicts a lack of FLASH effect in this modality. However, it is not clear whether the oxygen depletion model in other modalities is valid, or even has different characteristics.

Finally, the physico-chemical approach taken here is inherently flawed in that it does not take into account any of the biological processes. More specifically it does not acknowledge the possibility of differences in genomic makeup of tumour cells which are expressed due to long exposure to hypoxic conditions, diminishing the effect of FLASH-radiation. This could be important, as it would also provide microscopic disease, which does not reside in hypoxic volumes, with different susceptibility to FLASH-radiation.

It is important to stress that this paper does not aim to explain the FLASH-effect but rather provides a tool to include some of the effects in a treatment planning system such that the effects can be taken into account when devising a treatment or a laboratory experiment, avoiding the confusion inherent when working with degenerate systems.

## Author contributions statement

Frank Van den Heuvel: concept, mathematical coding, main corresponding author.

Anna Vella: Monte Carlo simulations

Francesca Fiorini: Monte Carlo simulations and providing the data from the LIBRA project.

Mark Brooke: theoretical/mathematical underpinning,

Mark Hill: mechanistic background—FLASH dosimetry

Anderson Ryan: FLASH Experiments

Tim Maughan: valuable conversations

Amato Giaccia: mechanistic background, suggestion on presentation.

All authors reviewed the manuscript and provided input in the writing.

## Additional information

Parts of the paper have been presented at ESTRO 2020, and ASTRO 2020 [37, 39]
